# Supplementary material for: Deletion of wfs1 Impairs Oligodendrocyte Precursor Cells Dorsal Distribution and Myelination Through the wfs1-hmgcs1 Axis in Zebrafish
Source: Biology (Basel). 2026 Aug 16;15(16):1402. doi: 10.3390/biology15161402 (PMC13510151; doi:10.3390/biology15161402)
Supplement: Supplementary file 1 [file biology-15-01402-s001.zip › File S1-Transcriptome sequencing data and differentially expressed genes (DEGs) in wfs1-- zebrafish at 5 dpf/2.KEGG/wfs1KOvsWT/UP/src/dre03440.html]

dre03440


- dre:554171

- Up regulated genes

554171\_1(1.55156529802644)

- dre:450081

- Up regulated genes

450081(1.15186882580158)

- dre:404608

- Up regulated genes

404608(1.6130895372485)

- dre:404608

- Up regulated genes

404608(1.6130895372485)

- dre:450081

- Up regulated genes

450081(1.15186882580158)

- dre:406721

- Up regulated genes

406721(1.14352228278224)

- dre:450081

- Up regulated genes

450081(1.15186882580158)

- dre:406721

- Up regulated genes

406721(1.14352228278224)

- dre:406487

- Up regulated genes

406487(7.45084509342341)

- dre:558923

- Up regulated genes

558923(6.88953216261849)

- dre:794038

- Up regulated genes

794038(1.51724035153863)

Close
